# Sobrevida de Pacientes Transplantados Cardíacos com Doença de Chagas Sob Diferentes Regimes de Imunossupressores Antiproliferativos

**DOI:** 10.36660/abc.20230133

**Published:** 2023-10-16

**Authors:** Silas Ramos Furquim, Luana Campoli Galbiati, Monica S. Avila, Fabiana G. Marcondes-Braga, Julia Fukushima, Sandrigo Mangini, Luis Fernando Bernal da Costa Seguro, Iascara Wozniak de Campos, Tania Mara Varejão Strabelli, Fernanda Barone, Audrey Rose da Silveira Amancio de Paulo, Luciana Akutsu Ohe, Mariana Cappelletti Galante, Fabio Antonio Gaiotto, Fernando Bacal

**Affiliations:** 1 Hospital das Clínicas Faculdade de Medicina Universidade de São Paulo São Paulo SP Brasil Instituto do Coração do Hospital das Clínicas da Faculdade de Medicina da Universidade de São Paulo, São Paulo, SP – Brasil

**Keywords:** Sobrevida, Transplante de Coração, Doença de Chagas

## Abstract

**Fundamento:**

A Doença de Chagas (DC) é uma causa importante de transplante cardíaco (TC). O principal obstáculo é a reativação da DC (RDC), normalmente associada a altas doses de imunossupressores. Estudos anteriores sugeriram uma associação do micofenolato de mofetila com aumento na RDC. No entanto, preditores de mortalidade são desconhecidos.

**Objetivos:**

Identificar os fatores de risco de mortalidade em pacientes com DC após o TC e o impacto do regime antiproliferativo sobre a sobrevida.

**Métodos:**

Estudo retrospectivo com pacientes chagásicos submetidos ao TC entre janeiro de 2004 e setembro de 2020, em protocolo de imunossupressão que priorizava o uso de azatioprina e sua mudança para micofenolato de mofetila em caso de rejeição. Realizamos regressão univariada para identificar preditores de mortalidade e comparamos sobrevida, rejeição, e evidência RDC entre os pacientes que usavam azatioprina, micofenolato de mofetila, e aqueles que mudaram de azatioprina para micofenolato (grupo “Mudança”) após a alta. Um valor de p<0,05 foi considerado estatisticamente significativo.

**Resultados:**

Foram incluídos 85 pacientes, 54,1% homens, idade mediana 49 (39-57) anos, e 91,8% com prioridade na lista de espera. Dezenove (22,4%) usavam azatioprina, 37 (43,5%) micofenolato de mofetila, e 29 (34,1%) trocaram a terapia; a sobrevida não foi diferente entre os grupos, 2,9 (1,6-5,0) x 2,9 (1,8-4,8) x 4,2 (2,0-5,0) anos, respectivamente; p=0,4. Não houve diferença na taxa de rejeição (42%, 73% e 59% respectivamente; p=0,08) ou de RDC (*T. cruzi* positiva na biópsia endomiocárdica 5% x 11% x 7%; p=0,7; uso benzonidazol 58% x 65% x 69%; p=0,8; PCR positiva para *T. cruzi* 20% x 68% x 42% respectivamente; p=0,1).

**Conclusões:**

Este estudo retrospectivo com pacientes com DC e TC não mostrou diferença na sobrevida entre os diferentes regimes antiproliferativos. O uso de micofenolato de mofetila não foi associado com taxas significativamente mais altas de RDC ou rejeição do enxerto nesta coorte. Novos ensaios randomizados são necessários para abordar essa questão.

## Figura Central

**Figure:**
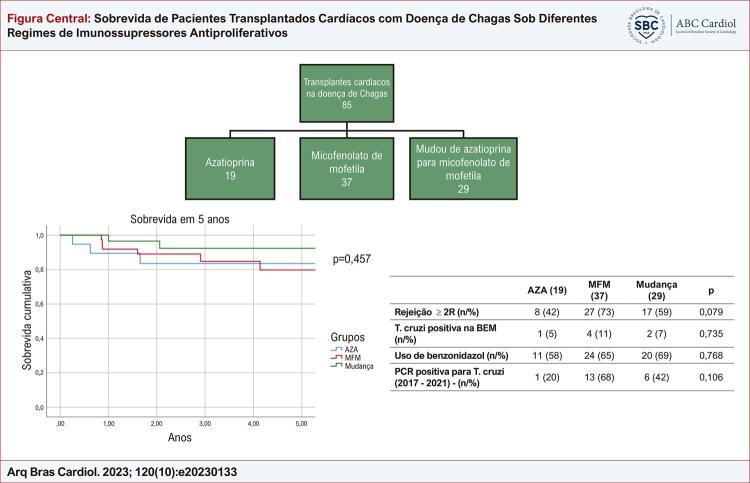
: Sobrevida de Pacientes Transplantados Cardíacos com Doença de Chagas Sob Diferentes Regimes de Imunossupressores Antiproliferativos

AZA: azatioprina; MFM: micofenolato de mofetila; BEMN: biópsia endomiocárdica.

## Introdução

A Doença de Chagas (DC), como etiologia da insuficiência cardíaca (IC), é um preditor independente de mortalidade na lista de espera para transplante cardíaco (TC).^1^ As principais complicações após o TC são infecções virais e bacterianas, rejeição do enxerto, câncer, e Reativação da DC (RDC).^2^ A RDC deve-se geralmente à imunossupressão excessiva, e ao uso de Micofenolato de Mofetila (MFM) em vez de Azatioprina (AZA).^3,4^ Assim, recomenda-se que pacientes com DC recebam terapia imunossupressora mais branda, com ciclosporina, AZA e esteroides, contanto que não ocorra rejeição.^5^

Embora existam registros frequentes da reativação da infecção pelo *Trypanosoma cruzi* (*T. cruzi*), essa é uma causa incomum de morte, dada à eficácia do tratamento da RDC com benzonidazol.^6-8^Propostas para reduzir o risco de reativação incluem redução de corticosteroides, níveis mais baixos de inibidores de calcineurina^8^ e uso preferencial de AZA.^3,4^

Apesar de evidências que corroboram o tratamento atualmente empregado, há poucos estudos que identificaram o impacto do uso da MFM versus AZA sobre a mortalidade, rejeição e RDC.^9^ Bacal et al.^3^ e Campos et al.^4^ conduziram duas análises de dados retrospectivos de pacientes com TC e DC, que receberam MFM imediatamente antes da cirurgia e encontraram que o medicamento foi associado com ocorrência aumentada de RDC, mas baixo risco de mortalidade.^3,4^ Outros estudos relataram risco aumentado de infecção por *T. cruzi* com o uso de MFM, mas eram limitados pelo tamanho amostral pequeno, por ser retrospectivo, e não especificar as doses de MFM utilizadas.^9,10^

Na população geral de pacientes com TC, o MFM reduz significativamente a mortalidade no primeiro ano em comparação à AZA,^11,12^ e mantém sua superioridade após um seguimento de três anos. No entanto, ainda existem perguntas não respondidas no manejo de pacientes com TC e DC, principalmente a ausência de evidência clínica sobre o regime imunossupressor para esses pacientes. Assim, nosso estudo tem como objetivo avaliar a sobrevida em longo prazo de pacientes com DC após TC em diferentes regimes antiproliferativos.

## Métodos

### Delineamento e população do estudo

Este é um estudo observacional, retrospectivo, que incluiu pacientes que se submeteram a TC por DC entre 01 de janeiro de 2004 e 30 de setembro de 2020.

Os pacientes foram divididos em três grupos, de acordo com o regime antiproliferativo: AZA (grupo AZA), MFM (grupo MFM) na alta hospitalar e aqueles que trocaram de AZA para MFM durante o seguimento (grupo “Mudança”).

Os critérios de exclusão foram óbito antes da alta, perda de seguimento, mudança de MFM para AZA e a ausência de uso de drogas antiproliferativas.

O estudo foi aprovado pelo comitê de ética institucional local (CAAE: 63584222.2.0000.0068).

### Fontes de dados

Os dados foram coletados a partir dos prontuários médicos institucionais, e de bancos de dados da farmácia e do departamento de doenças infecciosas.

### Imunossupressão

O esquema imunossupressor adotado consistiu na combinação tríplice padrão incluindo: esteroide, inibidor da calcineurina (ciclosporina ou tacrolimus) e antiproliferativo (AZA ou MFM) de acordo com protocolo da instituição. No grupo mudança, a AZA foi convertida para MFM quando indicado.

### Monitoramento e tratamento da reativação da infecção por T. cruzi

A infecção pelo T. cruzi foi investigada por biópsia endomiocárdica (BEM), realizada de acordo com o protocolo de TC (sete dias, 15 dias, três, seis e 12 meses após o TC ou na suspeita de rejeição do enxerto). A RDC também foi investigada em casos de evidência clínica de reativação, por reação em cadeia da polimerase do sangue periférico, desde 2017. Episódios suspeitos ou confirmados de RDC foram tratados com benzonidazol em uma dose de 5-10 mg/kg/dia por 60 dias.

### Análise estatística

Variáveis contínuas com distribuição normal foram expressas como média e desvio padrão, aquelas sem distribuição normal como mediana e intervalo interquartil, e a comparação entre os grupos foi realizada pelo teste t de Student não pareado ou teste de Mann-Whitney, respectivamente. As variáveis categóricas foram comparadas pelo teste do qui-quadrado e o teste exato de Fisher. A normalidade dos dados foi verificada pelo teste de Kolmogorov-Smirnov. Nos grupos de imunossupressão, a análise de sobrevivência foi realizada pelo método de Kaplan-Meier e o teste log-rank. Realizamos uma análise univariada pela regressão logística de Cox para identificar preditores de mortalidade. Valores de p<0,05 foram considerados estatisticamente significativos. As análises foram realizadas com o programa SPSS versão 26.

## Resultados

**Figura 1:**
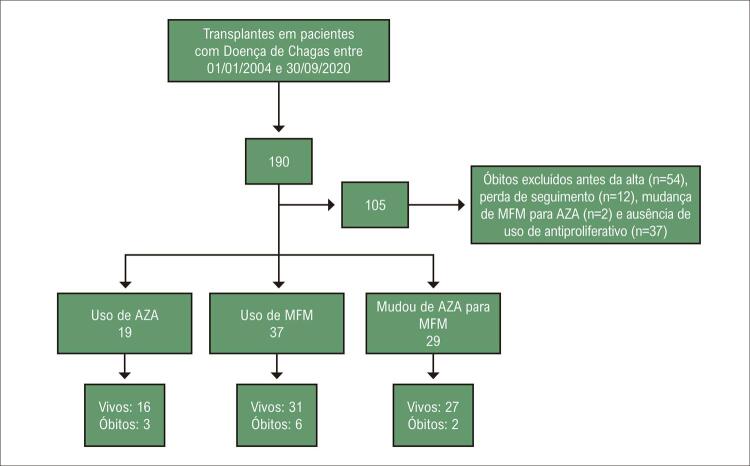
– Fluxograma da seleção de pacientes. AZA: azatioprina; MFM: micofenolato de mofetila.

Entre 01 de Janeiro de 2004 e 30 de setembro de 2020, 190 TC foram conduzidos em pacientes com DC. Foram excluídos das análises 105 pacientes – 54 morreram antes da alta, 12 perderam seguimento, dois mudaram de MFM para AZA e 37 não utilizaram drogas antiproliferativas (Figura 1).

Dezenove pacientes (22,4%) usaram AZA, 37 (43,5%) MFM, e 29 (34,1%) mudaram de AZA para MFM durante o acompanhamento. O tempo médio de uso de MFM foi 3,44 (±0,43) e 3,76 (±0,58) anos nos grupos MFM e “Mudança”, respectivamente. Não houve diferenças nas características basais entre esses grupos (Tabela 1). A duração mediana de seguimento foi 4,1 anos (IIQ: 1,6 -6,5) no grupo AZA, 2,9 (IIQ 1,8-6,0) no grupo MFM e 4,2 (IIQ: 2,0-6,1) no grupo Mudança (p = 0,336). A dose média diária de AZA foi 82,1 mg (±9,9), de MFM foi 962,2 mg (±57,6), e no grupo “Mudança” foi de 900,0 mg (±77,9). Não houve diferenças nas taxas de rejeição entre os grupos AZA, MFM e “Mudança” (42% x 73% x 59%, respectivamente p=0,08), positividade para *T. cruzi* na BEM (5% x 11% x 7%, respectivamente; p=0,7), PCR positiva para *T. cruzi* (20% x 68% x 42%, respectivamente; p=0,8), ou uso de benzonidazol (58% x 65% x 69%, respectivamente; p=0,1) (Tabela 2).

**Tabela 1:** – Características dos pacientes (azatioprina x micofenolato de mofetila x mudança de azatioprina para micofenolato de mofetila)

	AZA (19)	MFM (37)	MUDANÇA (29)	p
**Acompanhamento (anos)**	4,1 (1,6 -6,5)	2,9 (1,8-6,0)	4,2 (2,0-6,1)	0,336
**Idade do receptor (anos)**	48,6 (±9,3)	48,59 (±12,7)	45,0 (±11,8)	0,416
**Sexo (masculino)**	12 (63,2)	15 (40,5)	19 (65,5)	0,870
**Raça**				
Branca	11 (57,9)	24 (64,9)	12 (41,4)	
Não branca	8 (42,1)	13 (35,1)	17 (58,6)	0,158
**Peso do receptor (Kg)**	60,7 (±8,7)	62,2 (±1,8)	60,3 (±9,8)	0,738
**Altura do receptor (cm)**	164 (±1,9)	163 (±1,3)	166 (±1,6)	0,476
**Tipo sanguíneo**				
O	10 (52,6)	14 (37,8)	17 (58,6)	
A	7 (36,8)	15 (40)	8 (27,6)	
B	2 (10,5)	5 (13,5)	2 (6,9)	
AB	0 (00,0)	3 (8,1)	2 (6,9)	0,652
**Tempo na lista de espera (dias)**	50 (21-190)	56 (17-140)	41 (29-77)	0,347
**Intermacs**				
1	1 (5,3)	2 (5,4)	0 (0,0)	
2	3 (15,8)	15 (40,5)	11 (37,9)	
3	14 (73,7)	17 (45,9)	17 (58,6)	
4	1 (5,3)	3 (8,1)	1 (3,4)	0,307
**Droga vasoativa**	18 (94,7)	33 (89,2)	27 (93,1)	0,781
**Balão intra-aórtico**	9 (47,4)	24 (64,9)	21 (72,4)	0,199
**Uso de DAV**	0 (0,0)	1 (2,7)	1 (3,4)	1,000
**Hemodiálise pré-TC**	0 (0,0)	5 (13,5)	2 (6,9)	0,268
**Inibidor de calcineurina**				
Tacrolimo	10 (52,6)	27 (73,0)	23 (79,3)	
Ciclosporina	9 (47,4)	10 (27,0)	6 (20,7)	0,129

Valores em n (%), média (DP) ou mediana (intervalo interquartil). Intermacs: Interagency Registry for Mechanically Assisted Circulatory Support. TC: Transplante Cardíaco; DAV: dispositivo de assistência ventricular; AZA: azatioprina; MFM: micofenolato de mofetila.

**Tabela 2:** – Evidência de reativação da doença de Chagas em pacientes em uso de azatioprina ou micofenolato de mofetila e pacientes que mudaram o regime imunossupressor (grupo “mudança”)

	AZA (19)	MFM (37)	MUDANÇA (29)	p
Rejeição ≥ 2R	8 (42)	27 (73)	17 (59)	0,079
T. cruzi positivo na BEM	1 (5)	4 (11)	2 (7)	0,735
Uso de Benzonidazol	11 (58)	24 (65)	20 (69)	0,768
PCR positivo para T. cruzi (2017 – 2021)	1 (20)	13 (68)	6 (42)	0,106

Valores em n (%); T. cruzi: Trypanosoma cruzi; BEM: Biópsia Endomiocárdica; PCR: reação em cadeia da polimerase; AZA: azatioprina; MFM: micofenolato de mofetila.

Onze pacientes foram a óbito e 74 sobreviveram em cinco anos de seguimento. Na análise univariada, as características basais dos pacientes que foram a óbito não foram diferentes daquelas dos pacientes que sobreviveram (Tabela 3).

**Tabela 3:** – Características dos pacientes (óbitos x sobreviventes)

	Óbitos (n = 11)	Sobreviventes (n = 74)	Valores p	HR
**Agente antiproliferativo**				
Azatioprina	3 (15,8)	16 (84,2)		
Micofenolato de mofetila	6 (16,2)	31 (83,8)	0,995	0,99 (0,25-3,98)
Mudança de AZA para MFM	2 (6,9)	27 (93,1)	0,308	0,39 (0,07-2,36)
**Idade do receptor (anos)**	51,1 (±1,7)	42,8 (±1,7)	0,178	1,04 (0,98-1,10)
**Sexo (masculino)**	9 (81,8)	37 (50,0)	0,083	0,26 (0,06-1,19)
**Raça**				
Branca	7 (63,6)	40 (54,1)		
Afro-americana	4 (36,4)	34 (45,9)	0,468	0,63 (0,18-2,17)
**Peso do receptor (Kg)**	61,6 (±8,4)	61,16 (±10,1)	0,990	1,00 (0,94-1,06)
**Altura do receptor (cm)**	166,9 (±3,3)	163,5 (±0,9)	0,380	1,03 (0,96-1,11)
**Incompatibilidade de gêneros (doador-receptor)**	4 (36,4)	38 (51,4)	0,437	0,61 (0,18-2,10)
**Idade do doador (anos)**	29,4 (±0,94)	31,2 (±0,28)	0,490	1,03 (0,95-1,10)
**Tipo sanguíneo**				
O	7 (63,6)	34 (45,9)	0,649	
A	2 (18,2)	28 (37,8)	0,243	0,39 (0,08-1,89)
B	1 (9,1)	8 (10,8)	0,837	0,80 (0,1-6,56)
AB	1 (9,1)	4 (5,4)	0,744	1,42 (0,17-11,57)
**Tempo na lista de espera (dias)**	47 (19-90)	61 (32-163)	0,972	1,00 (0,99-1,00)
**Prioridade na lista**	10 (90,9)	68 (91,9)	0,975	1,03 (0,13-8,13)
**Intermacs**				
1	0 (0,0)	3 (4,1)	0,237	0,00 (-)
2	5 (45,5)	24 (32,4)	0,222	0,36 (0,069-1,86)
3	4 (36,4)	44 (59,5)	0,041	0,17 (0,31-0,93)
4	2 (18,2)	3 (4,1)	0,237	
**Droga vasoativa**	9 (81,8)	69 (93,2)	0,227	0,39 (0,08-1,80)
**Balão intra-aórtico**	6 (54,5)	48 (64,9)	0,562	0,70 (0,21-2,31)
**Uso de DAV**	0 (0,0)	2 (2,7)	0,778	0,05 (0,00-71784102,8)
**Hemodiálise pré-TC**	2 (18,2)	5 (6,9)	0,254	2,44 (0,53-11,33)
**Inibidor de calcineurina**				
Tacrolimo	7 (63,6)	53 (71,6)		
Ciclosporina	4 (36,4)	21 (28,4)	0,542	0,68 (0,20-2,33)
**Rejeição ≥ 2R**	6 (54,5)	46 (63,9)	0,568	1,41 (0,43-4,63)
**T. cruzi positivo na BEM**	2 (18,2)	5 (6,9)	0,280	2,33 (0,50-10,78)
**Uso de benzonidazol**	8 (72,7)	47 (63,5)	0,517	1,55 (0,41-5,85)
**PCR positivo para T. cruzi (2017 – 2021)**	3 (100)	17 (48,6)	0,379	64,77 (0,01-701814,7)

Valores em número (%), média (DP) ou mediana (intervalo interquartil); Intermacs: Interagency Registry for Mechanically Assisted Circulatory Support; DAV: dispositivo de assistência ventricular; TC: transplante cardíaco; T. cruzi: Trypanosoma cruzi; BEM: biópsia endomiocárdica; PCR: reação em cadeia da polimerase; AZA: azatioprina; MFM: micofenolato de mofetila.

### Taxas de sobrevida

**Figura 2:**
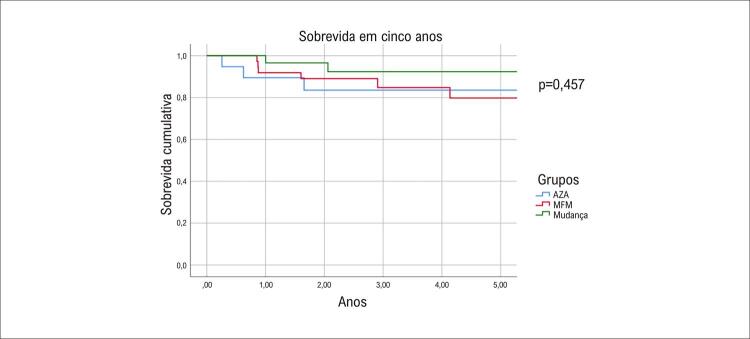
– Análise de sobrevida pelos métodos de Kaplan Meier e log-rank. AZA: azatioprina; MFM: micofenolato de mofetila.

As taxas de sobrevida em cinco anos não foram diferentes entre os grupos AZA, MFM e Mudança 2,9 (1,6-5,0) x 2,9 (1,8-4,8) x 4,2 (2,0-5,0) anos, respectivamente; p=0,457 (Figura 2). Os principais resultados estão resumidos na Figura Central.

## Discussão

Com base em estudos randomizados, o medicamento antiproliferativo de escolha após o TC tem sido o MFM.^1,12^ No entanto, em pacientes com DC, há evidências de estudos observacionais e retrospectivos indicando taxas mais altas de RDC com o uso de MFM, mas sem diferença significativa na sobrevida.^3,4,13^ O impacto da RDC sobre a mortalidade após o transplante não é conhecido e, por isso, o agente antiproliferativo de escolha tem sido AZA. Embora uma taxa mais alta de RCD era esperada nos pacientes em uso de MFM, com base em estudos prévios, nossos resultados não mostraram diferenças nas taxas de sobrevida em cinco anos entre pacientes usando AZA e MFM.

Vários métodos diagnósticos para RDC têm sido usados nos estudos; Bacal et al.^3^ utilizaram xenodiagnóstico e cultura sanguínea que pode ser positiva em pacientes com DC crônica, e Campos et al.^4^ não usaram PCR. Observamos uma taxa mais alta de uso empírico de benzonidazol, revelando a dificuldade diagnóstica da RDC na prática clínica. A maioria dos casos não apresentam sinas ou sintomas sugestivos, requerendo alta suspeição. No momento, não há um método diagnóstico preciso para RDC, o que reforça a necessidade de melhorar seu diagnóstico e monitoramento para melhor avaliar seu impacto em longo prazo sobre o TC. Neste cenário, a PCR pode ser uma ferramenta útil na monitorização da RDC. Benvenuti et al.^14^ sugerem reativação da DC se for detectada alta carga parasitária em um único exame de sangue ou após dois resultados de PCR positivos consecutivos de intensidade crescente. No caso de baixa carga parasitária, um PCR positivo na BEM é indicativo de RDC. No entanto, há uma grande discordância entre PCR no sangue periférico e BEM positiva, além de uma ausência de ponto de corte estabelecido para PCR.^14^

Apesar de uma porcentagem mais alta de rejeição nos grupos MFM e Mudança em comparação ao grupo AZA, não houve diferença estatística. A tendência de uma maior rejeição com o uso de MFM é contrária aos dados da literatura, em que o uso de MFM foi associado com menores taxas de rejeição e de mortalidade.^15^ Outro estudo prévio que avaliou o uso de MFM e AZA em pacientes com DC também não relatou diferença nas taxas de rejeição.^3^ Acreditamos que essa tendência possa estar relacionada com o delineamento do estudo, em que os pacientes nos grupos MFM e Mudança apresentaram, necessariamente, rejeição prévia. Isso justificou o uso de MFM em nossa instituição, o que, por sua vez, foi um fator de risco para novas rejeições.^16^ Ensaios clínicos randomizados maiores, de superioridade, são necessários para investigar se pacientes usando MFM apresentam maior sobrevida que pacientes usando AZA.

Em relação ao agente antiproliferativo de escolha e as taxas de RDC, não houve diferenças durante o acompanhamento entre os grupos de diferentes regimes imunossupressores. Observou-se uma alta taxa de evidência (clínica ou laboratorial) para RDC em todos os grupos. Outras características basais não foram diferentes entre os pacientes.

### Limitações

Apesar do número relativamente pequeno de pacientes, em nosso conhecimento, este é o maior estudo do tipo coorte analisando regimes imunossupressores em pacientes com DC submetidos a TC, e dados de ensaios randomizados não estão disponíveis ainda. Nosso estudo tem limitações, como seu delineamento retrospectivo e em único centro, ausência de um protocolo de pesquisa para RDC, uso recente de PCR para *T. cruzi* (2017), e considerável número de pessoas que perderam seguimento ou não usaram drogas antriproliferativas. Não avaliamos as causas de morte dos pacientes, o que poderia estar relacionado à rejeição ou RDC e forneceria mais informações sobre diferentes regimes imunossupressores. Também não avaliamos os critérios usados para o diagnóstico e o tratamento para RDC.

## Conclusão

Este estudo retrospectivo não encontrou diferença na sobrevida entre pacientes com DC após TC em diferentes regimes imunossupressores. O uso de MFM não foi estatisticamente associado a taxas mais altas de RDC ou rejeição do enxerto nesta coorte. Novos ensaios clínicos randomizados são necessários para abordar essa questão.
