# Supplementary material for: Sobrevida de Pacientes Transplantados Cardíacos com Doença de Chagas Sob Diferentes Regimes de Imunossupressores Antiproliferativos
Source: Arq Bras Cardiol. 2023 Oct 16;120(10):e20230133. [Article in Portuguese] doi: 10.36660/abc.20230133 (PMC10586812; doi:10.36660/abc.20230133)
Supplement: Translation

# Translation: Survival of Heart Transplant Patients with Chagas’ Disease Under Different Antiproliferative Immunosuppressive Regimens

**Authors:** Silas Ramos Furquim, Luana Campoli Galbiati, Monica S. Avila, Fabiana G. Marcondes-Braga, Julia Fukushima, Sandrigo Mangini, Luis Fernando Bernal da Costa Seguro, Iascara Wozniak de Campos, Tania Mara Varejão Strabelli, Fernanda Barone, Audrey Rose da Silveira Amancio de Paulo, Luciana Akutsu Ohe, Mariana Cappelletti Galante, Fabio Antonio Gaiotto, Fernando Bacal

**Affiliations:** 1 Brazil Instituto do Coração do Hospital das Clínicas da Faculdade de Medicina da Universidade de São Paulo, São Paulo, SP – Brazil

**Keywords:** Survival, Heart Transplantation, Chagas Disease

## Abstract

**Background:**

Chagas’ disease (CD) is an important cause of heart transplantation (HT). The main obstacle is Chagas’ disease reactivation (CDR), usually associated to high doses of immunosuppressants. Previous studies have suggested an association of mycophenolate mofetil with increased CDR. However, mortality predictors are unknown.

**Objectives:**

To identify mortality risk factors in heart transplant patients with CD and the impact of antiproliferative regimen on survival.

**Methods:**

Retrospective study with CD patients who underwent HT between January 2004 and September 2020, under immunosuppression protocol that prioritized azathioprine and change to mycophenolate mofetil in case of rejection. We performed univariate regression to identify mortality predictors; and compared survival, rejection and evidence of CDR between who received azathioprine, mycophenolate mofetil and those who changed from azathioprine to mycophenolate mofetil after discharge (“Change” group). A p-value < 0.05 was considered statistically significant.

**Results:**

Eighty-five patients were included, 54.1% men, median age 49 (39-57) years, and 91.8% were given priority in waiting list. Nineteen (22.4%) used azathioprine, 37 (43.5%) mycophenolate mofetil and 29 (34.1%) switched therapy; survival was not different between groups, 2.9 (1.6-5.0) x 2.9 (1.8-4.8) x 4.2 (2.0-5.0) years, respectively; p=0.4. There was no difference in rejection (42%, 73% and 59% respectively; p=0.08) or in CDR (T. cruzi positive by endomyocardial biopsy 5% x 11% x 7%; p=0.7; benznidazole use 58% x 65% x 69%; p=0.8; positive PCR for T. cruzi 20% x 68% x 42% respectively; p=0.1) rates.

**Conclusions:**

This retrospective study did not show difference in survival in heart transplant patients with CD receiving different antiproliferative regimens. Mycophenolate mofetil was not associated with statistically higher rates of CDR or graft rejection in this cohort. New randomized clinical trials are necessary to address this issue.

## Introduction

Chagas’ disease (CD), as etiology of heart failure (HF), is an independent predictor of mortality in the waiting list for heart transplantation (HT).^1^ Main complications after HT are bacterial and viral infections, graft rejection, cancer, and CD reactivation (CDR).^2^ CDR is usually due to excessive immunosuppression and the use of mycophenolate mofetil (MMF) instead of azathioprine (AZA).^3,4^ Therefore, it is recommended that patients with CD receive milder immunosuppressive therapy, with cyclosporine, AZA and steroids as long as there is no rejection.^5^

Even though there are frequent reports of reactivation of *Trypanosoma cruzi* (*T. cruzi*) infection, it is an unusual cause of death, due to the efficacy of CDR treatment with benznidazole.^6-8^ Proposals to reduce the risk of reactivation include early reduction of corticosteroids, lower serum levels of calcineurin inhibitors^8^ and preferential use of AZA.^3,4^

Despite the evidence supporting the currently employed treatment, few studies have identified the impact of the MMF versus AZA use on mortality, rejection and CDR.^9^ Bacal et al.^3^ and Campos et al.^4^ conducted two retrospective data analysis of heart transplant patients with CD in which MMF was administered right before the operation and found that the drug was related with increased CDR, but with low risk for mortality.^3,4^ Other studies reported increased risk of *T.cruzi* infection with the use of MMF, but they were limited by the small sample size, being retrospective and not specifying the MMF doses used.^9,10^

In the general heart transplant population, MMF reduces significantly mortality in the first year compared to AZA,^11,12^ and maintains its superiority after a three-year follow-up. However, there are still several unanswered questions in the management of HT patients with CD, especially a lack of clinical evidence on the ideal immunosuppressive regimen for these patients. Therefore, our study has the aim to evaluate the long-term survival of patients with CD after HT under different antiproliferative regimens.

## Methods

### Study design and population

This is a retrospective, observational study that included patients who had undergone HT due to CD between 01 January 2004 and 30 September 2020.

Patients were divided into three groups, according the antiproliferative regimen: AZA (AZA Group) and MMF (MMF group) on hospital discharge and those who changed from AZA to MMF during the follow-up (Change group). Exclusion criteria were death before discharge, loss of follow-up, change from MMF to AZA and non-use of antiproliferative drugs.

The study was approved by local institutional review board (CAAE: 63584222.2.0000.0068).

### Data source

Data were collected from institutional electronic medical records, as well as pharmacy and infectious diseases department databases.

### Immunosuppression

Immunosuppressive regimen consisted of a combination of three drugs: steroids, cyclosporine or tacrolimus and AZA or MMF, according to the institutional protocol. AZA was preferred as antiproliferative in patients with CD. In cases of graft rejection, AZA was converted to MMF.

### Monitorization and treatment of Trypanosoma cruzi infection reactivation

*Trypanossoma cruzi* infection was investigated by endomyocardial biopsy (EMB), performed according to the HT protocol (seven days, 15 days, three, six and 12 months after HT or in clinical suspicion of graft rejection). CDR has been investigated in cases of clinical evidence of reactivation by polymerase chain reaction (PCR) of the peripheral blood since 2017. Episodes of confirmed or suspected CDR were treated with benznidazole in a 5-10 mg/kg/day dosage for 60 days.

### Statistical analysis

Continuous variables with normal distribution were expressed as mean and standard deviation and those without normal distribution as median and interquartile range, and these were compared between the groups by unpaired Student’s t-test or Mann-Whitney test, respectively. Categorical variables were compared by chi-square and Fisher’s exact tests. Data normality was verified using Kolmogorov-Smirnov test. In the groups of immunosuppression, survival analysis was performed by the Kaplan-Meier method and the log-rank test. We performed a univariate analysis by Cox logistic regression to identify mortality predictors. P values <0.05 were considered statistically significant. Statistics were performed with SPSS software version 26.

## Results

From 01 January 2004 to 30 September 2020, 190 HT were performed in patients with CD. One hundred and five patients were excluded from analysis: 54 died before discharge, 12 were lost to follow-up, 2 changed MMF to AZA and 37 did not use antiproliferative drugs (Figure 1).

**Figure 1:** – Flowchart of patients’ selection. AZA: azathioprine; MMF: mycophenolate mofetil.

Nineteen (22.4%) patients used AZA, 37 (43.5%) MMF and 29 (34.1%) changed from AZA to MMF during the follow-up. The mean time of MMF use was 3.44 (±0.43) and 3.76 (±0.58) years in MMF and Change groups, respectively. There were no differences in baseline characteristics between these groups (Table 1). Median follow-up duration was 4.1 (IQR: 1.6 -6.5) years in the AZA group, 2.9 (IQR: 1.8-6.0) in MMF group and 4.2 (IQR: 2.0-6.1) in Change group (p = 0.336). Mean daily AZA dose was 82.1 mg (±9.9), MMF was 962.2 mg (±57.6), and Change was 900.0 mg (±77.9). There were no differences between AZA, MMF and Change groups in rejection rates (42% x 73% x 59%, respectively; p=0.08), positivity to *T. cruzi* on EMB (5% x 11% x 7%, respectively; p=0.7) positive PCR for *T. cruzi* (20% x 68% x 42%, respectively; p=0.8) or benznidazole use (58% x 65% x 69%, respectively; p=0.1. Table 2).

**Table 1:** – Characteristics of patients (Azathioprine x Mycophenolate x Change)

	AZA (19)	MMF (37)	CHANGE (29)	p
**Follow up (years)**	4.1 (1.6 -6.5)	2.9 (1.8-6.0)	4.2 (2.0-6.1)	0.336
**Recipient age (years)**	48.6 (±9.3)	48.59 (±12.7)	45.0 (±11.8)	0.416
**Gender (male)**	12 (63.2)	15 (40.5)	19 (65.5)	0.870
**Race**				
White	11 (57.9)	24 (64.9)	12 (41.4)	
Nonwhite	8 (42.1)	13 (35.1)	17 (58.6)	0.158
**Recipient weight (Kg)**	60.7 (±8.7)	62.2 (±1.8)	60.3 (±9.8)	0.738
**Recipient height (cm)**	164 (±1.9)	163 (±1.3)	166 (±1.6)	0.476
**Blood type**				
O	10 (52.6)	14 (37.8)	17 (58.6)	
A	7 (36.8)	15 (40)	8 (27.6)	
B	2 (10.5)	5 (13.5)	2 (6.9)	
AB	0 (00.0)	3 (8.1)	2 (6.9)	0.652
**Time in waiting list (days)**	50 (21-190)	56 (17-140)	41 (29-77)	0.347
**Intermacs**				
1	1 (5.3)	2 (5.4)	0 (0.0)	
2	3 (15.8)	15 (40.5)	11 (37.9)	
3	14 (73.7)	17 (45.9)	17 (58.6)	
4	1 (5.3)	3 (8.1)	1 (3.4)	0.307
**Vasoactive drug**	18 (94.7)	33 (89.2)	27 (93.1)	0.781
**Intra-aortic balloon**	9 (47.4)	24 (64.9)	21 (72.4)	0.199
**VAD use**	0 (0.0)	1 (2.7)	1 (3.4)	1.000
**Hemodialysis pre-HT**	0 (0.0)	5 (13.5)	2 (6.9)	0.268
**Calcineurin Inhibitor**				
Tacrolimus	10 (52.6)	27 (73.0)	23 (79.3)	
Cyclosporine	9 (47.4)	10 (27.0)	6 (20.7)	0.129

Values are n (%), mean (SD) or median (interquartile range). Intermacs: interagency registry for mechanically assisted circulatory support. VAD: ventricular assist device; HT: heart transplantation; AZA: azathioprine; MMF: mycophenolate mofetil.

**Table 2:** – Evidence of Chagas disease reactivation in patients under azathioprine or mycophenolate mofetil regimen and patients that changed immunosuppression regimens

	AZA (19)	MMF (37)	CHANGE (29)	p
Rejection ≥ 2R	8 (42)	27 (73)	17 (59)	0.079
T. cruzi positive in EMB	1 (5)	4 (11)	2 (7)	0.735
Benznidazole use	11 (58)	24 (65)	20 (69)	0.768
Positive PCR for T. cruzi (2017 – 2021)	1 (20)	13 (68)	6 (42)	0.106

Values are n (%). T. cruzi: Trypanosoma cruzi; EMB: endomyocardial biopsy; PCR: polymerase chain reaction; AZA: azathioprine; MMF: mycophenolate mofetil.

There were 11 deaths and 74 survivors in five years of follow-up. In the univariate analysis, baseline characteristics of the patients who died were not different from those who survived (Table 3).

**Table 3:** – Characteristics of patients (dead x survivors)

	Dead (n = 11)	Live (n = 74)	p value	HR
**Antiproliferative**				
Azathioprine	3 (15.8)	16 (84.2)		
Mycophenolate	6 (16.2)	31 (83.8)	0.995	0.99 (0.25-3.98)
Change AZA to MMF	2 (6.9)	27 (93.1)	0.308	0.39 (0.07-2.36)
**Recipient age (years)**	51.1 (±1.7)	42.8 (±1.7)	0.178	1.04 (0.98-1.10)
**Gender (male)**	9 (81.8)	37 (50.0)	0.083	0.26 (0.06-1.19)
**Race**				
White	7 (63.6)	40 (54.1)		
African American	4 (36.4)	34 (45.9)	0.468	0,63 (0.18-2.17)
**Recipient weight (Kg)**	61.6 (±8.4)	61.16 (±10.1)	0.990	1.00 (0.94-1.06)
**Recipient height (cm)**	166.9 (±3.3)	163.5 (±0.9)	0.380	1.03 (0.96-1.11)
**Donor-recipient gender mismatch**	4 (36.4)	38 (51.4)	0.437	0.61 (0.18-2.10)
**Donor age (years)**	29.4 (±0.94)	31.2 (±0.28)	0.490	1.03 (0.95-1.10)
**Blood Type**				
O	7 (63.6)	34 (45.9)	0.649	
A	2 (18.2)	28 (37.8)	0.243	0.39 (0.08-1.89)
B	1 (9.1)	8 (10.8)	0.837	0.80 (0.1-6.56)
AB	1 (9.1)	4 (5.4)	0.744	1.42 (0.17-11.57)
**Time in waiting list (days)**	47 (19-90)	61 (32-163)	0.972	1.00 (0.99-1.00)
**Priority on the list**	10 (90.9)	68 (91.9)	0.975	1.03 (0.13-8.13)
**Intermacs**				
1	0 (0.0)	3 (4.1)	0.237	0.00 (-)
2	5 (45.5)	24 (32.4)	0.222	0.36 (0.069-1.86)
3	4 (36.4)	44 (59.5)	0.041	0.17 (0.31-0.93)
4	2 (18.2)	3 (4.1)	0.237	
**Vasoactive drug**	9 (81.8)	69 (93.2)	0.227	0.39 (0.08-1.80)
**Intra-aortic balloon**	6 (54.5)	48 (64.9)	0.562	0.70 (0.21-2.31)
**VAD use**	0 (0.0)	2 (2.7)	0.778	0.05 (0.00-71784102.8)
**Hemodialysis pre-HT**	2 (18.2)	5 (6.9)	0.254	2.44 (0.53-11.33)
**Calcineurin inhibitor**				
Tacrolimus	7 (63.6)	53 (71.6)		
Cyclosporine	4 (36.4)	21 (28.4)	0.542	0.68 (0.20-2.33)
**Rejection ≥ 2R**	6 (54.5)	46 (63.9)	0.568	1.41 (0.43-4.63)
**T. cruzi positive in EMB**	2 (18.2)	5 (6.9)	0.280	2.33 (0.50-10.78)
**Benznidazole use**	8 (72.7)	47 (63.5)	0.517	1.55 (0.41-5.85)
**Positive PCR for T. cruzi (2017 – 2021)**	3 (100)	17 (48.6)	0.379	64.77 (0.01-701814.7)

Values are n (%), mean (SD) or median (interquartile range). Intermacs: interagency registry for mechanically assisted circulatory support; VAD: ventricular assist device; HT: heart transplantation; T. cruzi: Trypanosoma cruzi; EMB: endomyocardial biopsy; PCR: polymerase chain reaction; AZA: azathioprine; MMF: mycophenolate mofetil.

### Survival rates

Median survival rates in five years were not different between the AZA, MMF and Change groups 2.9 (1.6-5.0) x 2.9 (1.8-4.8) x 4.2 (2.0-5.0) years, respectively; p=0.457 (Figure 2). The main results are summarized in the Central Illustration.

**Figure 2:** – survival analysis by Kaplan Meier and Log-Rank. AZA: azathioprine; MMF: mycophenolate mofetil.

## Discussion

Based on randomized studies, the antiproliferative agent of choice after HT has been the MMF.^1,12^ Nonetheless, in patients with CD, there has been evidence from observational and retrospective studies indicating higher CDR rates with MMF use, although without significant difference in survival.^3,4,13^ The impact of CDR on mortality after transplantation is unknown, so the antiproliferative agent of choice has been AZA. Although a higher CDR rate was expected in patients using MMF based on previous studies, our results did not show different survival rates in five years between patients using AZA and MMF.

Various CDR diagnostic methods have been used in the studies; Bacal et al.^3^ used xenodiagnosis and blood culture that can be positive in patients with chronic CD, and Campos et al. did not use PCR.^4^ We observed a higher rate of empirical use of benznidazole, revealing the difficult to diagnose CDR in clinical practice; most cases do not present suggestive signs and symptoms, requiring high suspicion. At the present, there is no accurate CDR diagnostic method, which reinforces the need to improve its diagnosis and monitoring to better assess its long-term impact on HT. In this scenario, PCR may be a useful tool to monitor CDR. Benvenuti et al.^14^ suggest reactivation of CD if high parasitic load (PL) is detected in a single blood test or after at least two sequential positive PCR results of increasing intensity. In cases of low PL, a positive PCR on EMB is indicative of CDR. However, there is a great discordance between PCR in peripheral blood and positive EMB, in addition to lack of an established PCR cutoff.^14^

Despite a higher percentage of rejection in the MMF and Change groups compared to the AZA group, there was no statistical difference. The tendency of higher rejection with MMF use is contrary to literature data, where MMF use has been associated with lower rejection and mortality rates.^15^ Another previous study that evaluated MMF and AZA regimens in CD patients also found no difference in rejection rates.^3^ We believe that this tendency may be related to the study design, in which patients in the MMF and Change groups necessarily had a previous rejection. This justified the use of MMF in our institution, which in turn was a risk factor for new rejections.^16^ Larger, blinded, superiority randomized clinical trials should be done to investigate whether patients taking MMF have higher survival as compared with those using AZA.

Regarding the antiproliferative agent choice and CDR rates, there were no differences during the follow-up between the immunosuppressive regimen groups. There was a high rate of CDR evidence (clinical or laboratorial) in all groups. Other baseline characteristics were not different between the patients.

### Limitations

Despite the relatively small number of patients, to our knowledge, this study is the largest cohort study analyzing immunosuppressive regimens in CD patients undergoing HT, and data from prospective and randomized trials are not available yet. Our study has limitations such as its retrospective and single center design, lack of a research protocol for CDR, recent use of PCR for *T. cruzi* (2017), and considerable number of patients who were lost to follow-up or who did not use antiproliferative drugs. We did not evaluate patients’ causes of death, which could be related to rejection or CDR and could provide more information about different immunosuppressive regimens. We also did not assess the criteria used to diagnose and indicate treatment for CDR.

## Conclusion

This retrospective study did not find differences in survival of CD patients following HT between immunosuppressive regimens. The use of MMF was not statistically associated with higher rates of CDR or graft rejection in this cohort. New randomized clinical trials are necessary to address this issue.
